# Gene duplication and paleopolyploidy in soybean and the implications for whole genome sequencing

**DOI:** 10.1186/1471-2164-8-330

**Published:** 2007-09-19

**Authors:** Jessica A Schlueter, Jer-Young Lin, Shannon D Schlueter, Iryna F Vasylenko-Sanders, Shweta Deshpande, Jing Yi, Majesta O'Bleness, Bruce A Roe, Rex T Nelson, Brian E Scheffler, Scott A Jackson, Randy C Shoemaker

**Affiliations:** 1 Department of Agronomy, Purdue University, West Lafayette, IN 47907, USA; 2 Purdue Genetics Program, Purdue University, West Lafayette, IN 47907, USA; 3 Department of Chemistry and Biochemistry, University of Oklahoma, Norman, OK 73019, USA; 4 USDA-ARS, Corn Insect and Crop Genetics Research Unit, and Department of Agronomy, Iowa State University, Ames, IA 50011, USA; 5 USDA-ARS-MSA Genomics Laboratory, Stoneville, MS 38776, USA

## Abstract

**Background:**

Soybean, *Glycine max *(L.) Merr., is a well documented paleopolyploid. What remains relatively under characterized is the level of sequence identity in retained homeologous regions of the genome. Recently, the Department of Energy Joint Genome Institute and United States Department of Agriculture jointly announced the sequencing of the soybean genome. One of the initial concerns is to what extent sequence identity in homeologous regions would have on whole genome shotgun sequence assembly.

**Results:**

Seventeen BACs representing ~2.03 Mb were sequenced as representative potential homeologous regions from the soybean genome. Genetic mapping of each BAC shows that 11 of the 20 chromosomes are represented. Sequence comparisons between homeologous BACs shows that the soybean genome is a mosaic of retained paleopolyploid regions. Some regions appear to be highly conserved while other regions have diverged significantly. Large-scale "batch" reassembly of all 17 BACs combined showed that even the most homeologous BACs with upwards of 95% sequence identity resolve into their respective homeologous sequences. Potential assembly errors were generated by tandemly duplicated pentatricopeptide repeat containing genes and long simple sequence repeats. Analysis of a whole-genome shotgun assembly of 80,000 randomly chosen JGI-DOE sequence traces reveals some new soybean-specific repeat sequences.

**Conclusion:**

This analysis investigated both the structure of the paleopolyploid soybean genome and the potential effects retained homeology will have on assembling the whole genome shotgun sequence. Based upon these results, homeologous regions similar to those characterized here will not cause major assembly issues.

## Background

The vast majority of flowering plants likely have a polyploid origin [1-3]. The homeologous chromosomal regions resulting from these large-scale duplication events are subject to a wide range of structural changes including accumulation of indels [4,5], illegitimate recombination [6,7], gene loss, rearrangements, gene duplications and nucleotide divergence [8]. In addition, they are also subject to gene conservation [8]. Analyses of homeologous regions in maize provids clear evidence of fractionation following duplication [5,7,9,10]. However, this is not clearly the case for cotton. An analysis of homologous regions in cotton found extensive genic and intergenic conservation with differences found only in transposable elements and small indels [11].

Soybean (*Glycine max *(L.) Merr.) was characterized early as an ancient polyploid through genetic mapping studies that identified homeologous chromosome regions based upon duplicate RFLP markers [12-14]. In addition to mapping studies, analysis of BAC-end sequences has suggested that the retained duplicate regions of the soybean genome still share sequence homeology [15,16]. Similarly, hybridization based approaches showed fairly extensive sequence identity between RFLP anchored paralogous BACs [17,18]. Approximately 275 duplicate genes were identified in the soybean EST collections and estimates of synonymous distances between gene pairs suggested that soybean has undergone at least two rounds of large-scale duplication at approximately 14 and 42 million years ago (Mya)[19,20]. Although the origin of the duplications giving rise to homeologous genes is difficult to determine [21] it was assumed that they arose through large-scale duplication events such as polyploidy. Cytogenetic studies have shown that the 'diploid' *Glycine *have 2n = 40 chromosomes while other papilionoids have 2n = 10 or 11 suggesting at least one large-scale genome duplication [22]. In addition, segmental duplications in soybean were observed using fluorescence in situ hybridization (FISH)[23] and a more recent FISH analyses reveals near chromosomal-level homeology along chromosome 19 (linkage group L) and another unidentified chromosome, with only a few instances of disrupted colinearity [24].

Limited sequence comparisons have been conducted from homeologous regions of the soybean genome. Schlueter et al. [25] compared BAC sequences containing ω-6 fatty acid desaturase (FAD2) genes and found extensive gene conservation in both order and orientation between two BACs from homeologous regions with only one large inversion to distinguish their structures. Another study involving homeologous regions containing an N-hydroxycinnamoyl/benzoyltransferase (HCBT) gene cluster gave similar results with nucleotide identity between most genes upwards of 95% [8]. These high levels of sequence identity between homeologous regions have been suggested as a potential source of error during whole genome shotgun sequence assembly in a paleopolyploid species.

Recently, the DOE-JGI and the USDA jointly announced that the soybean genome was to be sequenced through a whole-genome shotgun (WGS) approach [26]. Since little is known about the structure, organization, similarity and full extent of the duplications within the soybean genome, questions remain about the efficacy of a resulting assembly of these sequences. In this study, we identified, sequenced and characterized 11 BAC clones representing 5 distinct homeologous regions of the genome. In addition, 6 BACs previously characterized for homeology were included [8,25] in the assembly analysis for a total of 17 BAC clones representing 7 homeologous soybean genomic regions. This collection of BACs was identified as containing genes that anchor potential homeologous regions of the genome. Duplicate genes were identified from ESTs by using TBLASTX and building contigs as previously described [25]. Each new "anchor gene" was chosen due to a related role in seed development of soybean. Duplicate BACs were sequenced and analyzed to determine the amount of genic homeology. In addition, the ability to distinguish homeologous sequences as will be expected for assembly of WGS was evaluated by merging sequence traces for all 17 BACs and ressemblying with varying parameters. Each assembly was evaluated against the original individual BAC assemblies. Our results indicate that the paleopolyploid soybean genome is a mosaic of homeologous sequences ranging from instances of high gene conservation to regions with extremely limited conservation. Except for tandem duplications and long simple sequence repeats, adequate nucleotide differences exist between even the most conserved homeologous regions to completely distinguish them during sequence assembly.

## Results

### Duplicate soybean BACs: sequencing, assembly and homeology

Shotgun sequencing of 17 soybean BACs selected for containing retained duplicate loci yielded a total of 36,873 sequence traces and a total of 2,028,159 bp of assembled soybean genomic sequence (Table 1). Six BACs (768,449 bp) have previously been shown to represent homeologous regions of the soybean genome anchored by either N-hydroxycinnamoyl/benzoyltransferase genes (HCBT; gmw1-74i13 and gmw1-52d3; [8] or ω-6 fatty acid desaturase genes (FAD2; gmw1-105h23, gmw1-15k6, gmw1-11j16, gmw1-45m6; [25]. The 11 additional sequenced BACs were anchored by either RFLP clones (A711; UMb001-24d13 and Umb001-5f5) or by the duplicate transcripts cellulose synthase (gmw2-133d1 and gmw1-93l19), galactinol synthase (gmw1-5g16 and gmw1-103e11), raffinose synthase (gmw1-13o17 and gmw1-8g7) and caffeoyl-CoA O-methyltransferase (gmw1-58k3, gmw1-57d24 and gmw1-27d20). To date, this is the largest analysis of homeologous regions from the soybean genome. Although most of the BACs were sequenced to completion (phase III), seven remaining BACs contained a small number of ordered contigs with fewer than three gaps (phase II) and one BAC (gmw1-27d20) was phase I with five ordered contigs (Table 1).

**Table 1:** General BAC information

								Ratio^e ^of			
BAC	Linkage group	Genbank accession	SNP ID^b^	Length (bp)	Phase	Gap	ORFs^c^	Average^d ^EST coverage	EST- based coverage	Overall gene homeology^f^	Gene density^g^

gmw2-133d1	F	AC158503	8001	117591	III	0	13	32.6	38.2	3 of 13	1/9.05
gmw1-93l19	M	AC166092		51037	III	0	5	62.4	50.5	3 of 5	1/10.2
gmw1-105h23	O	AC187294	30491	134287	III	0	18	82.0	76.4	18 of 18	1/7.46
gmw1-15k6	I	AC160454	26051	148858	III	0	22	77.0	71.1	18 of 22	1/6.77
gmw1-11j16	L	AC166091		69947	III	0	9	82.2	83.0	2 of 9	1/7.77
gmw1-45m6	^a^	AC166742		143028	III	0	7	53.6	53.0	1 of 7	1/20.4
gmw1-5g16	O	AC169184		115953	II	2	11	74.0	68.8	4 of 11	1/9.66
gmw1-103e11	I	AC166090		89397	III	0	12	78.6	81.3	4 of 12	1/7.45
gmw1-58k3	O	AC185959		177331	II	2	8	50.7	47.5	3 of 8	1/22.2
gmw1-57d24	D1a	AC170860	20113	162359	II	2	19	75.0	71.5	3 of 19	1/9.02
gmw1-27d20	D1b	AC173959	16079	227022	I	6	24	65.4	61.9	3 of 24	1/9.46
gmw1-74i13	C1	DQ336954	5981	173654	III	0	18	68.3	70.4	13 of 18	1/9.65
gmw1-52d3	C2	DQ336955		98675	III	0	10	59.2	62.1	9 of 10	1/9.87
gmw1-13o17	D1a	AC196857		89030	II	5	9	41.5	48.0	1 of 9	1/11.1
gmw1-8g7	^a^	AC196858		53292	III	0	4	32.6	30.7	1 of 4	1/13.3
UMb001-24d13	E	DQ347960	13567	111223	II	1	8	84.0	79.3	3 of 8	1/13.9
UMb001-5f5	A2	DQ347961	42937	65475	II	2	5	91.9	94.6	3 of 5	1/10.9
Average				119303			14	59.1	59.05		1/11.1

^a ^Unmappable; no polymorphic SSRs identified or any matches of CDS to SNP data

^b ^SNP Ids are taken directly from Choi et al. (2007). EST sequence from which SNP derived found in Methods and Materials.

^c ^Does not include ORFs that are alternatively spliced

^d ^An average across the BAC of the number of bp supported by an EST or cDNA divided by the total number of bp for each annotation

^e ^A ratio of the total number of bp on the BAC that are annotated divided by the total number of bases that have EST or cDNA support

^f ^Count is based upon the number of homeologs shared between BACs out of the total number of genes

^g ^Gene density is in 1 gene per × number of kilobases

With the exception of BACs UMb001-24d13 and Umb001-5f5 that were already mapped by an RFLP marker (A711), all but two of the remaining BACs were mapped by either BLAST-based identity of predicted coding sequence (CDS) to previously mapped transcript-based single nucleotide polymorphisms (SNPs) [27] or simple sequence repeats (SSRs) identified from each BAC sequence. Eight SNP markers were identified. Six of these markers confirmed already known map positions for gmw1-105h23, gmw1-15k6 [25], gw1-74i13 [8], UMb001-24d13, UMb001-5f5 (RFLP marker A711) and gmw2-133d1 (mapped by SSR as described below). The final two SNPs provided map positions for gmw1-57d24 and gmw1-27d20 (Table 1). In addition to SNPs, SSRs derived from BACs were identified, tested for polymorphisms and mapped. Only two BACs, gmw1-8g7 and gmw1-45m6 showed no polymorphisms in the mapping population or any matches to mapped transcript-based SNPs [25]. Although there are multiple BACs on linkage groups I and O, eleven linkage groups are represented in this analysis (Table 1).

A total of 238 genes were predicted across the ~2.03 Mb of soybean sequence for an average gene density of 1 gene/11.1 Kb (Table 1) slightly less than previous estimates [28,29,8,25]. All gene structure predictions as well as the annotations, *ab initio *predictions and EST-based support for each structure can be viewed at the following website [30]. On average, 59.06% of the predicted gene structures had either EST or cDNA based support, regardless of whether coverage was normalized for gene size (average EST coverage) or not (ratio of EST coverage; Table 1).

**Figure 1:**
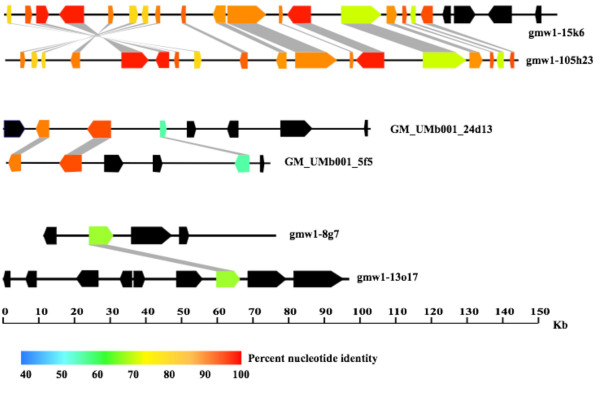
**Summary of genic conservation from putative homeologous BACs in soybean**. Duplicate genes from six soybean BACs (3 different pairs) show the range of gene conservation found in the soybean genome. Each block-arrow represents a predicted gene structure. Black arrows are genes with no homeolog. Colored arrows are genes with a homeolog. A heat map for percent nucleotide identity shows the average nucleotide identity between duplicate genes for each conserved homeolog. Gray boxes between structures show homoelogous relationships. All gene structure predictions are available online [30]. The first BAC pair has been reprinted with permission from The Plant Genome [19].

Levels of gene conservation between BACs varied from being gene for gene in both order and orientation, with the exception of an eight-gene block inversion, for BACs gmw1-15k6 and gmw1-105h23 [25] to very weak homeology anchored by only a single gene (gmw1-13o17 and gmw1-8g7; Table 1; Figure 1). While both of these extremes were observed, more often, homeologous BACs showed mid-range homeology; i.e. approximately 25 to 50% of genes in overlapping regions are retained. In those cases, most retained homeologs had 90% or greater sequence identity (Table 2) with a few extremes. The average nucleotide identity between homeologs ranged from 53.7 to 97.4% with an average of 86.6% while average protein similarity ranging from 53.3 to 99.0% with an average of 88.8% (Table 2). It should be noted that when homeologs were also tandemly duplicated on a BAC, they were not included in these estimates due to the inability to accurately determine which gene copy was the true ancestral homeolog between BACs.

**Table 2:** Duplicate gene homeology/paralogy between BAC pairs

BAC homeologs	Putative function	# of exons	Coding length^a^	Nucleotide identity	Protein identity	Protein similarity	Ks	Ka	Date (Mya)
gmw1-74i13 gmw1-52d3	^b^	^b^	^b^	89.8	88.0	90.7	0.1490	0.0335	12.2

gmw1-105h23 gmw1-15k6	^d^	^d^	^d^	90.7	88.9	90.4	0.1061	0.0326	8.70

UMb001-24d13	DNA binding	6	1338	92.7	88.7	92.2	0.1177	0.0468	9.65
UMb001-5f5	DNA binding	7	1473						
UMb001-24d13	Gamma response I	9	987	95.9	95.7	96.3	0.1405	0.0152	11.52
UMb001-5f5	Gamma response I	9	984						
UMb001-24d13	Selenium binding	4	1881	56.3	54.6	56.4	0.1709	0.0575	14.01
UMb001-5f5	Selenium binding	5	585						

gmw1-103e11	*A. thaliana*-like NAP	7	510	96.4	95.8	97.2	0.0933	0.0188	7.65
gmw1-5g16	*A. thaliana*-like NAP	7	1002						
gmw1-103e11	Beta-fructofuranosidase	6	1944	94.4	92.7	94.1	0.0716	0.0276	5.87
gmw1-5g16	Beta-fructofuranosidase	6	1956						
gmw1-103e11	Galactinol synthase	4	732	90.5	93.5	94.7	0.3208	0.0316	26.30
gmw1-5g16	Galactinol synthase	3/4	669/987						
gmw1-103e11	RAD-like protein	6/7	564/900	96.9	92.9	97.6	0.0432	0.0442	3.54
gmw1-5g16	RAD-like protein	5	240						

gmw2-133d1	GTPase	14	3183	96.9	98.1	99.1	0.1055	0.0084	8.65
gmw1-93l19	GTPase	16	3480						
gmw2-133d1	Cellulose synthase	9	2211	67.6	65.1	67.0	0.1109	0.0438	9.09
gmw1-93l19	Cellulose synthase	5	924						
gmw2-133d1	Chain A protein	1	1608	81.1	76.4	80.1	0.1856	0.077	15.21
gmw1-93l19	Chain A protein	1	1452						

gmw1-13o17	Raffinose synthase	5	2277	66.4	71.5	81.5	2.5495	0.2051	208.98
gmw1-8g7	Raffinose synthase	6	2190						

gmw1-57d24	Phospholipase C	8	1308	80.5	78.7	87.6	0.5457	0.114	44.73
gmw1-58k3	Phospholipase C	8	1299						
gmw1-57d24	COMT	5	747	79.7	79.0	88.3	0.6442	0.1204	52.80
gmw1-58k3	COMT	4/5	615/354						

gmw1-58k3	COMT	4/5	615/354	73.6	76.3	87.7	1.7076	0.1667	139.97
gmw1-27d20	COMT	5	744						
gmw1-58k3	Otubain	6	1992	53.7	42.5	53.3	4.024	0.3023	329.84

gmw1-27d20	Otubain	7	1860						
gmw1-57d24	CBS	6/8	399/687	74.9	73.7	89.5	2.0095	0.1562	164.71
gmw1-27d20	CBS	8	678						
gmw1-57d24	COMT	5	747	74.1	81.6	91.0	1.5875	0.1196	130.12
gmw1-27d20	COMT	5	744						

	**Average**			***86.6***	***85.4***	***88.8***	***0.4239***	***0.0577***	***34.75***
	*Recalculated average 1*^d^			*89.8*	*88.2*	*90.1*	*0.1179*	*0.0341*	*9.665*
	*Recalculated average 2*^e^			*71.8*	*71.9*	*82.7*	*1.8668*	*0.1691*	*153*

^a ^Coding length in base pairs based upon CDS (from start to stop not including introns).

^b ^The values for homeologs between gmw1-74i13 and gmw1-52d3 are previously reported (Schlueter et al. 2006). Identity, similarity, Ks, Ka and Dates shown are average across BACs.

^c ^The values for homeologs between gmw1-105h23 and gmw1-15k6 are previously reported (Schlueter et al. 2007). Identity, similarity, Ks, Ka and Dates shown are average across BACs.

^d ^Recalculated average not including the highly divergent homeologs from gmw1-13o17, gmw1-8g7, gmw1-57d24, gmw1-58k3 and gmw1-27d20.

^e ^Recalculated average for just the highly divergent homeologs from gmw1-13o17, gmw1-8g7, gmw1-57d24, gmw1-58k3 and gmw1-27d24.

To visualize the level of nucleotide identity between BACs, VISTA plots for BACs anchored by the RFLP A711, cellulose synthase, galactinol synthase, raffinose synthase and caffeoyl-CoA O-methyltransferase (COMT) were generated [see Additional Files 1, 2, 3, 4, 5]. VISTA identity plots as well as values for nucleotide identity, protein identity and protein similarity for HCBT and FAD2-anchored BACs have been previously reported [8,25 respectively]. Nucleotide identity between BACs is strongest in the coding regions and extends both 5' and 3' from predicted genes before dropping to below 50% between BACs with more duplicate gene conservation [8,25]. This is likely due to retained non-coding sequences such as promoter elements between homeologous regions. However, as the level of gene conservation drops, so does the nucleotide identity beyond duplicate genes.

In a number of cases, homeologs appear to have varying gene lengths such as the selenium-binding protein found on BACs UMb001-24d13 and UMb001-5f5 (Figure 1, third homeolog) [see Additional file 1]. The exon number for this gene varies and a stop codon in the first exon of the UMb001-24d13 encoded selenium-binding protein truncates the resulting transcript (Table 2). There is however, EST-based support for the mRNA on UMb001-24d13 extending further 3' but the alignment is not a perfect match (92% identity). Other cases of variation in exon number between duplicate genes are observed (Table 2). Most of the differences can be accounted for in two ways: 1) *ab initio *based prediction of gene structures with little to no EST support vary between BACs and/or 2) truncation of one of the predicted genes due to an encoded stop codon. Reliance on *ab initio *predictions for gene structures combined with the lack of EST-based support can lead to differences between homeologs in exon number. In many cases, even alignment to putative orthologs could not verify the gene structure.

Synonymous (Ks) and nonsynonymous (Ka) substitutions between all of the duplicate genes were calculated (Table 2). The average Ks value was 0.42398 and average Ka value was 0.05775. Again, the Ks and Ka values for HCBT and FAD2 BACs are previously reported [8,25]. All Ks values gave an average divergence estimate of 34.75 Mya. This value likely is inflated due to the extensive divergence between the duplicate genes identified on gmw1-57d24, gmw1-58k3 and gmw1-57d24 and between raffinose synthase on gmw1-13o17 and gmw1-8g7. When these duplicate genes were excluded from the calculation, the average divergence estimate was 9.665 Mya, similar to previous estimates [25] but still more recent than EST-based estimates [19,20]. When only the most divergent duplicate genes are used for coalescence estimates, a date of 153 Mya was obtained. Two caveats to divergence estimates should be noted: 1) The Ks values for the most divergent duplicate genes were for the most part well past saturation (greater than 1) and 2) in the most divergent regions, we cannot be certain that we are comparing homeologs and not paralogs (segmental or single gene duplications) without the context of the whole genome or more sequence in these regions. Only two pairs of homeologs showed evidence for positive selection; a ribonuclease HII encoding gene on gmw1-15k6 and gmw1-105h23 with a Ka/Ks ratio of 2.078 [25] and the RAD-like encoding gene from gmw1-103e11 and gmw1-5g16 with a Ka/Ks ratio of 1.023. All other retained homeologs appear to be under purifying selection for retained function.

### Reassembly of paleoduplicate regions

To quantify the potential confounding effects of paleopolyploidy on soybean whole-genome shotgun sequence assembly (WGS), all of the sequencing traces for the 17 BACs discussed above were used in large-scale or batch assemblies. The goal was to determine what effect homeology between duplicated regions will have as the soybean genome is reconstructed. Base-calling and assemblies were performed using Phred and Phrap, respectively [31-33] with default parameters and viewed in Consed [34].

**Figure 2:**
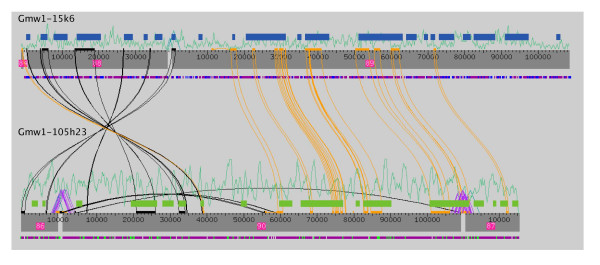
**Reassembly of highly identical homeologous soybean BACs**. Output of Phred/Phrap batch re-assembly of traces from gmw1-105h23 and gmw1-15k6 as viewed using Consed. Grey boxes represent the assembled contigs and are scaled in base pairs across each contig. Contig numbers are shown in pink boxes and are arbitrarily assigned by Phred/Phrap during sequence assembly. The blue and green boxes above each assembly show the predicted gene positions for gmw1-15k6 and gmw1-105h23, respectively. The green line-plot above each contig shows the average clone pair consistency. Sequence matches within and between contigs were determined with Cross-Match as part of Consed. Black lines within and between contigs show sequence matches that are in reverse orientation, while the orange lines show sequence matches in the same orientation. The bars between sequence matches correspond to the length of the match. Purple peak-shaped lines between contigs show clone pairs that span a gap. Below each contig is a purple line containing either blue (gmw1-15k6) or green (gmw1-105h23) tick marks; these are the tags that distinguish between traces from each BAC.

To first test if standard assembly parameters could distinguish between the most conserved homeologous BACs, sequence trace files for gmw1-105h23 and gmw1-15k6 were combined into a single "batch" assembly. Figure 2 shows that there is no cross assembly and no inclusion of sequencing traces between BACs. Assemblies were analyzed both manually and based upon BAC-specific tags to determine that sequence traces were assembled into the correct BAC contig. There are obvious regions with high levels of sequence identity between the BACs as determined by Crossmatch (Figure 2). Even with upwards of 97% sequence identity in exonic regions, sequence traces resolved into their correct "original" BACs. Quantification of the "batch-based" reassemblies against the original single-BAC assemblies was done using Vmatch [35]. The three reassembled contigs for gmw1-105h23 had 99.58% sequence identity with 99.06% coverage to the original BAC assembly. Likewise, for gmw1-15k6 the resulting reassembly contigs had 99.80% sequence identity with 99.44% sequence coverage. As these results show, the assemblies were nearly identical to the original BAC assembly with the exception of small sequence gaps between the contigs, although clone pair ends clearly order and orient the contigs (Figure 2). Extrapolated to a whole-genome scale assembly, this shows that for soybean, unless there are regions of the genome that have higher levels of homeology than has been observed, the conserved paleopolyploidy of soybean will not have a substantial effect on the genome assembly.

All of the 38,673 traces from all 17 BACs were then combined into a single assembly using both standard assembly parameters as well as various other parameter sets. Assemblies were quantified using three measures: 1) the number of contigs containing greater than 100 traces versus the original 35 contigs from individual BAC assemblies 2) average percent coverage of the reassembled contigs to original contigs and 3) average percent nucleotide identity of the reassembled contigs to the original contigs (Table 3). These last two values were determined by Vmatch analysis that performed a global pair-wise alignment between all of the reassembled contigs and original assembly contigs as described in materials and methods. Under all of the parameter sets, some contigs were split into multiple contigs thereby increasing the contig number to greater than the original 35.

**Table 3:** Assessment and quantification of reassembly of duplicate BAC sequences

Assembly number	Parameters	Total # contigs	# contigs (> 100)^a^	% Coverage of old contigs^b^	% Identity to old contigs^c^	% Coverage +103e11^d^	% Identity +103e11^d^
1	standard	551	44	98.52%	99.07%	98.44%	97.39%
2	revise_greedy	2538	45	91.41%	99.08%	92.74%	98.43%
3	forcelevel 5	2140	40	96.13%	99.21%	95.56%	98.52%
4	minmatch 30	2184	50	94.77%^e^	98.92%^e^	95.51%	97.91%
5	forcelevel 3	2326	43	98.40%	98.60%	97.74%	97.96%
6	forcelevel 5 minmatch 30	1781	43	88.75%^e^	99.18%^e^	86.17%	98.04%
7	forcelevel 3 minmach30	1950	46	93.38%^f^	99.18%^f^		

^a ^Total number of contigs that contain greater than 100 sequence traces

^b ^Total length of the resulting contigs (not including any overlapping regions) divided by the length of the originally assembled BAC

^c ^Percent identity as calculated from Vmatch

^d ^Recalculated percent coverage and percent identity to include contigs containing traces from gmw1-103e11; these contigs did not meet the 80% sequence identity cutoff for Vmatch

^e ^One contig from gmw1-103e11 met the cutoff criteria of 80% sequence identity for Vmatch and was included in this estimation. The second contig was included in the +103e11 calculations

^f ^This parameter set matches the parameter set that was determined to give the best reassembly of gmw1-103e11 as a single BAC reassembly. Both resulting contigs met the 80% sequence identity cutoff for Vmatch and are included in these averages.

Experimental parameters were varied in an attempt to increase the percent coverage and percent nucleotide identity of the batch assemblies. The first parameter, revise_greedy, split initial contig assemblies at weak joins (regions that may be misassembled between duplicate regions due to sequence identity) and then attempted to reattach them for a higher overall alignment score. While only barely increasing the percent identity score, the percent coverage score was reduced by just over 7%. The forcelevel flag specifically reduced the stringency during the final contigs merge pass with 0 being most stringent and 10 least stringent, standard parameters using 0. When the forcelevel was relaxed slightly to 3, the percent coverage was nearly the same with only a slight drop in percent identity. However, increasing forcelevel to 5 decreased the percent coverage by just over 2% but increased the percent identity by over a full percent. It also had the effect of reducing the number of contigs from 44 at forcelevel 0 to 40 at forcelevel 5. Finally, the minmatch value was adjusted from 14 (standard) to 30 to increase the assembly stringency, a modification that dramatically increased the number of contigs to 50, as expected, and dropped the overall percent coverage. Combinations of these parameter changes also were investigated and the results are given as assemblies 6 and 7. Overall, it appears that standard Phred/Phrap assembly parameters return the greatest percent coverage out of all assemblies as well as the nearly best percent identity to the original contig assemblies.

### Sources of potential assembly errors

Two potential sources of assembly error were identified in this analysis. First, under the last three assembly conditions (assemblies 5–7, Table 3) a contig from gmw1-27d20 and from GM_UMb-5f5 were incorrectly merged at a large (TATA)_n _simple sequence repeat region. The resulting contig clearly shows the transition from one BAC to the other across the TA repeat with low quality sequences and low sequence coverage flanking the repeat. Lower quality sequences are not uncommon with simple sequence repeats that are large in length as these regions are difficult to sequence through. Secondly, the assembly of BAC gmw1-103e11 was especially troublesome in both the "batch" assembly of all of the BACs and on an individual assembly scale. Table 3 shows how the inclusion of the 103e11 contigs (which in most cases did not meet the Vmatch parsing criteria as is noted in Table 3) lowers both the average percent coverage and percent identity across the assembly.

Under standard assembly conditions, the 89,397 bp BAC gmw1-103e11 is fragmented into two contigs, a 19,452 bp contig with clone pair matches to the middle of the larger 69,905 bp contig. Clearly, a region from the middle of gmw1-103e11 is misassembled into a separate contig. This region can be partially resolved without manual reassembly by changing the forcelevel to 3 and minmatch to 30. The assembly still results in two contigs, but this is due to a gap in the middle of the contig and not exclusion of a region in the middle of the contig as with standard assembly parameters. The overall sequence coverage is 84.7% and sequence identity of 82.49% to the original BAC sequence. When this parameter set is used to reassembly all of the BACs however, it reduces the percent coverage by just over 5% but does increase the percent identity by almost 2% (Table 3).

**Figure 3:**
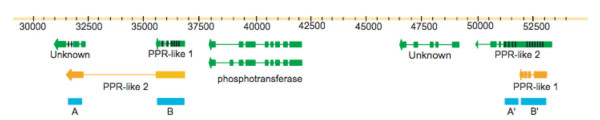
**Repetitive sequences in BAC gmw1-103e11**. Gene positions and repetitive sequences found in the region of 30,000 bp to 53,000 bp on gmw1-103e11. Predicted gene structures are shown as green boxes and arrows, with the boxes representing exons and lines being introns. Black tick marks on a gene show the start position of a repeated PPR domains within the gene. The blue boxes show the repetitive sequences identified by Vmatch. Orange gene alignments reflect the realignment of predicted gene structures back to the genomics sequence.

This then raised the question as to what in the gmw1-103e11 sequence could be causing the re-assembly (both individual BAC and in the context of all BACs) to generate a second contig from the middle of the BAC. Utilizing Vmatch to identify sequence matches within the region being misassembled, non-retroelement, highly identical unique repeats (blue rectangles on Figure 3) were identified. Two major repeats occur in tandem in this region; a 566 bp repeat that is 96% identical (labelled as A and A' on Figure 3) and a 1, 198 bp repeat that is 95% identical (labelled as B and B' on Figure 3). Repeat A is present in the first unknown gene, repeat B in the pentatricopeptide repeat (PPR)-like 1 gene and both of the secondary repeat copies, A' and B' are contained within the PPR-like 2 gene (Figure 3).

GeneSeqer alignments [36] were generated of each predicted gene structure from this region realigned to the gmw103e11 BAC sequence. A portion of the PPR-like 2 gene aligns to the region predicted to contain the PPR-like 1 and unknown genes (Figure 3; orange gene structures). Similarly, the PPR-like 1 gene aligns to a portion of PPR-like 2. All of these alignments were using the "moderate" stringency function of GeneSeqer. The two predicted PPR-like genes in this region vary greatly in their structures and lengths. As discussed above, often there is little to no EST support and *ab initio *predictions must be relied upon. For this region, the first unknown gene has 7 ESTs with only 90% sequence identity that support the last exon, the rest of the gene is based upon *ab initio *predictions. The phosphotransferase and second unknown gene have nearly full EST support. Both of the PPR-like genes, however, are completely *ab initio *predicted.

Although there is variation in the predicted structures of the PPR-like genes, BLASTP annotation identified conserved petatricopeptide repeat (PPR) repeats in both. PPR repeats are a degenerate ~30 amino acid motif that occur tandemly multiple times within a protein [37]. To identify potential PPR repeats across this region, MEME and MAST were used to generate PPR motifs and search the gmw1-103e11 BAC sequence for all possible occurrences of the motif [38]. Two PPR repeats were found in the first intron of the predicted unknown gene, at least six PPR repeats were identified in the PPR-like 1 gene and eleven repeats were identified in the PPR-like 2 gene. These PPR repeats are 81–99 nucleotides in length that range from 25–100% similar at the amino acid level and 33–95.8% similar at the nucleotide level (within and between both PPR-like genes). The black lines on Figure 3 show the start location of the PPR domains that are located end to end within the coding sequence. These repeats account for the Vmatch identified repeat sequences A/A' and B/B'. The similarity of a portion of PPR-like 2 to both the first unknown gene and PPR-like 1 suggests two scenarios: 1) PPR-like 2 is incorrectly predicted and should be two separate genes or 2) PPR-like 2 is incorrectly predicted and should be fused with the first unknown gene. In either case, these PPR containing genes and repeats are the source of assembly error, as discussed below.

Identified repeats A/A', B/B' and all of the predicted genes from this region of gmw1-103e11 were re-aligned using GeneSeqer to the Phred/Phrap re-assembled gmw1-103e11 contigs. Both of the PPR-like gene structure predictions as well as the repeat A containing unknown gene align to a ~3,500 bp region in the middle of the 69,905 bp major contig. This region also contains clone pair matches to both ends of the 19,452 bp secondary contig. What has occurred is the PPR-containing regions are above the threshold of distinguishing one copy from another and have collapsed into a single structure in the larger contig. The phosphotransferase gene and second unknown gene are excluded from this region and placed in the separate contig. These results show that highly identical tandemly duplicated genes, especially those genes that themselves contain repetitive domains will be a potential source of assembly errors. In this case, the structure of the PPR repeats across the PPR-like genes cannot be resolved without manual curation of the assembly.

### Composition of whole-genome shotgun sequence assembly

**Figure 4:**
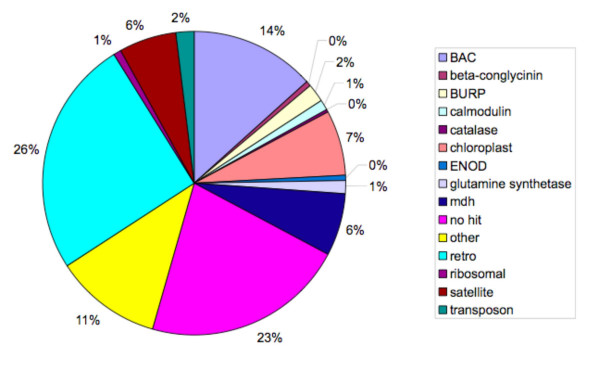
**Sequence composition of highly represented sequences in a small-subset of JGI sequence traces**. A pie-chart representation of repetitive sequences from assembly of 80,000 JGI soybean whole-genome shotgun trace files. BAC corresponds to any contig that showed greatest identity to already assembled soybean BAC sequence. Mdh refers to a previously sequenced region of soybean containing repetitive sequence. No hit means that there was no blast-based match to the nonredundant database. Other was a best match to a sequence (BAC or genomic) from another organism that was not characterized. Satellite refers to known Sb92 or Str120 centromeric repeat sequences. The rest of the categories are as described in the figure legend.

To determine how well our assemblies were screening for highly repetitive sequence, a preliminary assembly using standard Phred/Phrap parameters of 80,000 randomly chosen JGI trace files was done. Contigs containing greater than 15 traces were considered highly represented even after initial trace screening against known repetitive sequences. Each of these contigs was subject to a BLAST-based annotation against the NCBI nonredundant database and then clustered into groups based upon that annotation (Figure 4). Surprisingly, 23% of the JGI contigs showed no sequence identity to any anything in the NCBI nonredundant database. However, when the contigs comprising this 23% are BLASTed against the repetitive database generated by Gill et al. [39] only 5 contigs out of 44 had no match and 7 contigs had a bit-score less than 90 and were considered poor matches. Forty thousand randomly chosen JGI trace files were combined with the 36,978 BAC generated trace files in a standard Phred/Phrap assembly. The addition of the JGI whole-genome shotgun generated trace files had no effect on either the percent identity of the reassembled contigs (99.07%) or on the percent coverage (98.52%).

## Discussion

In this analysis, we have characterized homeologous sequences from the paleopolyploid soybean genome and studied the effect of conserved duplicate regions on sequence assembly. Identified BACs map to 11 of the 20 soybean linkage groups representing a broad sampling of potential homeologous regions across the soybean genome. Previous analyses have shown fairly extensive sequence conservation between homeologous blocks in soybean [8,25]. Sequenced BACs identified as containing transcribed duplicate genes show a range of gene conservation (Figure 1; Additional Files 1, 2, 3, 4, 5: Supplemental Figures 1, 2, 3, 4).

Early analysis of the structure and organization of a paleopolyploid genome have been in maize. The "maize model" suggests that the present maize genome is a result of extensive reciprocal deletions as well as major transposable element insertions causing genome expansion and contraction resulting in homeologous regions that are not well conserved [5,7,9,10]. Conversely, in cotton, a relatively recent allotetraploid, the homologs studied were highly conserved with only small indels and transposable element insertions differing between regions [11]. The "cotton model" suggests strong duplicate gene conservation that extends well into the intergenic regions. In this analysis we find that the soybean genome is a mosaic of these two models with a range of conservation spanning from gene for gene retention [25] to moderately conserved regions with 25 to 50% gene retention [8] and highly divergent regions with a single gene conserved (Figure 1).

Coalesence estimates suggest that the most of the regions diverged approximately 9.6 Mya. This value falls within the range of what has previously been observed [8,25]. On the extreme end, however, five BACs contain highly divergent duplicate genes. These may indeed be the result of gene translocation, segmental or single gene duplication and not the result of polyploidy. While in the absence of the whole genome sequence we cannot be certain of the mechanism by which these genes duplicated, some support for at least a larger duplication event is found from the genetic map. Mapping of duplicate RFLP markers in soybean provided early evidence for a major genome duplication event [12]. Utilizing the most recent genetic map [27], linkage groups D1a and D1b (where gmw1-57d24 and gmw1-27d20 map, respectively) were found to contain an RFLP A725 that is duplicated between these linkage groups. In addition, D1b and O (where gmw1-27d20 and gmw1-58k3 map, respectively) both contain the RFLP K011 duplicated between linkage groups. While the linkage positions of these markers are separated by many centimorgans (data not show), it does lend credence to these linkage groups having a shared ancestry. A similar comparison for gmw1-13o17 and gmw1-8g7 could not be done because gmw1-8g7 is unmapped. Regardless of the mechanism, in soybean, there are regions of paleoduplicated chromosomes that have diverged greatly since duplication while others have not (Figure 1) [see Additional files 1, 2, 3, 4, 5].

Size differences between duplicate genes were observed on many of the BACs (Table 2). Even though on average 59% of the predicted genes had some EST support, the reliance on *ab initio *predictions results in variation between duplicate genes in gene structure predictions. A similar issue is observed with the PPR-like genes on gmw1-103e11 that are a potential source of batch assembly error. In addition, the varying levels of protein identity in homeologous regions may be the result of unsupported gene structure predictions. This analysis clearly shows that for improved annotation of the whole genome assembly, more transcript (EST, cDNA, etc.) sequences will be necessary to verify predicted gene structures.

Most plant genome sequencing efforts have been BAC-based using highly inbred plants with pseudo-monoploid genomes (diploid or polyploid plants with identical paleoduplicated genomes). As a result, plant genome assemblies have not been confounded by the effects of retained homeology in paleopolyploid regions of the genome. Conversely, many of the non-plant eukaryotic sequencing efforts have been WGS such as *Fugu rubripes *[40], mouse [41,42], and the Celera version of the human genome [43,44] to name only a few. Comparisons between the WGS project and BAC-based sequencing project in humans have found that while the WGS provides more accurate gene coverage more quickly, the BAC-based sequencing has much better coverage of repetitive sequences, especially highly conserved repeats and in the long run is more accurate in both order and orientation of genes [44-47]. A somewhat similar comparison between the *Oryza sativa *L. ssp. *indica *[48] and *Oryza sativa *L. ssp. *japonica *[49] sequencing projects concluded that the major differences in sequence assemblies are due to regions with large transposable elements [50].

The soybean genome is a well-documented paleopolyploid [12,51] as are all sequenced plants, e.g., Arabidopsis [52], rice [48,49,53,54] and most recently Poplar [55]. Although homeologous blocks could be identified in each of these species, even the most recent polyploidy events are thought to be more ancient than what has been described in soybean [19,20]. The often high levels of sequence conservation in homeologous regions in soybean [8,25] has raised the question of what effect this will have on the assembly of the whole-genome shotgun sequence effort (WGS) currently underway.

The reassembly of 17 homeologous BACs in soybean provides the first look at the effects a relatively conserved paleopolyploid genome on WGS assembly. The most identical homeologous BACs sequenced, gmw1-105h23 and gmw1-15k6 are just under 95% identical across both the BAC coding and noncoding regions (Table 2) [25]. Reassembly of these two BACs showed no misassembly of the BACs and no cross-assembly of trace files from one BAC in the other BAC (Figure 2). In the context of the WGS assembly, this is good news for homeologous regions that share less than 95% sequence identity. Under standard assembly parameters using Phrep/Phrap, paleoduplicate homeologous regions should be resolvable.

When all 17 BACs are reassembled in batch, observed assembly errors are the results of tandem duplications and simple sequence repeats. Analysis of the re-assembled BAC gmw1-103e11 shows that tandem duplications of genes such as the PPR-like genes with sequence identity greater than 95% may cause assembly issues. Using a standard set of parameters, clone pairs cannot be distinguished, especially when the repeat is larger than the sequence reads (generally over 500 bp). The parameter set that better resolves tandem repeats may not be the appropriate parameter set for all assemblies; as a result, hand assembly of these regions may be necessary for completion of genome assembly. Similarly, large simple sequence repeats may cause incorrect merging of regions. It should be noted however, if there are homeologous regions of the soybean genome that are conserved with greater than 95% sequence identity, they will likely behave in a manner similar to tandem duplications and may be more difficult to distinguish.

What was not observed in the batch reassembly was errors caused by retrotransposon sequences. In soybean, many of the potential retrotransposons have not been characterized although a number of studies are underway to identify repetitive sequences in soybean Marek et al. unpublished results [39]. This analysis, with one exception, did not identify BACs that contained numerous repetitive sequences; instead they were found to be gene rich. BAC gmw1-45m6 [25] does contain numerous LTR retrotransposons, but re-assembly of this BAC showed few errors. Cytogenetic studies have shown that the high-copy sequences in soybean are highly concentrated to centromeric and pericentromeric regions [24,56]. In addition, ongoing analysis of repetitive sequence in soybean shows that it is primarily in the centric, telomeric and nucleolar organizing regions of the genome (Gill et al. unpublished results) [26]. Contrary to maize or some species of rice [10,57], no evidence for a large burst of retrotransposon activity has been found in soybean. It is likely then, that in the context of WGS assembly, retrotransposon sequences in most cases will not affect assembly of genic regions.

Preliminary analysis of contigs generated from JGI trace files give an estimation of what repetitive sequences will need to be screened for during WGS assembly (Figure 4). Even though the 80,000 JGI traces were prescreened against characterized soybean repeats, those trace files that contain a fragment of a repeat are passing through the screening process. Further, there are enough sufficient sequences that assemble to regenerate the original repetitive sequence into a contig, or at least enough of the sequence to match back to characterized repeats. One previously noted consequence of WGS assembly is that the exclusion of transposable element sequences and repetitive sequences during assembly has the effect of eliminating genes that might be found in these regions [45]. In this case, genic sequences that flank or are contained in repetitive regions may be able to pass through the repeat screening such that they become part of the assembly. A balance between screening for repetitive sequences during WGS assembly while not excluding genic information will need to be found.

## Conclusion

This analysis has shown that the soybean genome is a mosaic of sequence conservation models for a paleopolyploid genome with some regions retaining all duplicate genes while other regions retain only one divergent duplicate gene. With this in mind, a study to determine how paleopolyploidy would affect whole genome shot-gun sequence assembly was undertaken. Our results have shown that even the most conserved homeologous BACs with upwards of 95% sequence identity show no cross-assembly (inclusion of sequence traces from one BAC into the other BAC). In addition, potential sources of assembly error were identified as tandem duplications with greater than 95% sequence identity and large simple sequence repeats.

## Methods

### Identification, sequencing and single BAC assembly of duplicate BACs

BACs gmw1-74i13 and gmw1-52d3, corresponding to duplicate loci anchored by N-hydroxycinnamoyl benzoyltransferase (HCBT) genes, were identified, sequenced and annotated by Schlueter [8]. Four BACs, gmw1-15k6, gmw1-105h23, gmw1-11j16 and gmw1-45m6 anchored by ω-6 fatty acid desaturase (FAD2) genes were identified, sequenced and annotated by Schlueter [25]. BACs anchored by the RFLP probe A711 with known cytogenetic information [24]. GM_UMb-24d13 and GM_UMb-5f5 were used to construct shotgun libraries for sequencing and assembly as described previously [56].

Retained duplicate transcripts corresponding to isoflavone synthase/cellulose synthase, galactinol synthase, raffinose synthase and caffeoyl-CoA o-methyltransferase were identified with TBLASTX (default parameters) using a reference sequence against all soybean ESTs [58]. Identified ESTs were aligned into contigs using Sequencher v.4.5, also with default parameters (Gene Codes Corp., MI). PCR primers were designed to distinguish between copies using Oligo 6.82 (Molecular Biology Insights, Cascade, CO) [see Additional file 6]. Multidimensional pools of the Williams 82 *G. max *BAC library (gmw1) were PCR screened. BAC DNA was isolated using a Plasmid Midi kit (Qiagen, Valencia CA) and reverified with PCR as previously described [8].

BACs gmw1-13o17 and gmw1-8g7 were subcloned and assembled as described in Schlueter [8]. Subclones were sequenced at the Iowa State DNA Sequencing and Synthesis Facility (Ames, Iowa). Sequence for BACs gmw2-133d1, gmw1-93l19, gmw1-5g16, gmw1-103e11, gmw1-58k3, gmw1-57d24 and gmw1-27d20 was generated at the University of Oklahoma using conditions previously described [59-63]. Accession numbers for all sequenced BACs can be found in Table 1.

### Mapping of duplicate BACs

BACs were mapped using two methods. First, already mapped EST-based SNPs were identified by BLASTN of annotated genes from each BAC against mapped ESTs [10]. Only ESTs that match to BAC-derived genes with an e-value of 0.0 (near identical match) were considered. In addition, each EST was aligned to the BAC to confirm that it corresponded to one homeolog (or paralog) versus the other. Secondly, each BAC that was not previously mapped was scanned for di- and tri-nucleotide repeats using Sputnik (Espresso Software Development, Seattle WA). Primer pairs flanking the potential SSR markers were designed using Oligo 6.82 (Molecular Biology Insights) and tested against various soybean parents of mapping populations. PCR reactions were 10 μl in volume and contained 1 × PCR buffer, 1.5 mM magnesium chloride, 5 mM dNTPs, 0.5 μM each primer, 50 ng *Glycine max *parental DNA, and 0.025 U of Taq DNA polymerase (Invitrogen). PCR cycling conditions were 94°C for 2 min, 35 cycles of 94° for 45 sec, 60° for 30 sec, 72° for 45 sec, followed by a final extension of 72° for 3 min. Resulting bands were run on either a 3% agarose 1 × TAE (Tris, Acetic Acid, EDTA) gel for larger (greater than 250 bp) products or 6% polyacrylamide 0.5 × PBE gel for smaller fragments. Polymorphic SSRs from each BAC were mapped in the *Glycine max *A81-356022 X *Glycine soja *PI 468.916 population [64,13]. Genetic map positions of these SSRs were determined using MapMaker/Exp 3.0 with a minimum lod score of 3.0 [64,65]. Sequences for these SSRs are available [see Additional file 7].

### Annotation of BACs

Gene prediction was done using a combination of ab initio and EST-alignment based methods as previously detailed [8,25]. Annotation was completed using yrGATE and viewed as part of the xGDB system [66,67]. A database with annotations was created called GmaxGDB [30]. Each predicted gene was subjected to a BLASTP query of the NCBI nr database with default parameters to assign a putative function. An e-value threshold of 1^e-10 ^was used to assign putative function.

### Determination of homeologs and divergence estimates

Alignment of homeologous BACs used shuffle-LAGAN [68] with default parameters anchored by predicted gene structures producing a VISTA plot [69]. The nucleotide and protein percent identity and similarity of homeologs, was calculated using WATER, a pairwise alignment program (gap penalty of 10; extension penalty of 0.2; EMBOSS)[70]. Synonymous and nonsynonymous distances were calculated using PAML, default parameters [71]. Coalesence estimates were calculated as in [20].

### Batch sequence assembly and quantification of assemblies

Trace files for all of the assembled BACs were combined into a single assembly utilizing 36,978 sequence reads. Base calling and sequence assemblies were performed using the Phred [31,32] and Phrap [33], respectively. Assemblies were viewed using the Consed viewer and Cross-Match [34]. All assemblies were run with standard Phred/Phrap parameters unless otherwise noted in the text or table. Briefly, parameters that were varied were: 1) revise_greedy that splits initial contig assemblies at weak joins (regions that may be misassembled due to high sequence identity) and then attempts to reattach them for a higher overall alignment score. 2) forcelevel reduces the stringency during the final contig merge pass and 3) minmatch which is the minimum length of a matching word in sequence comparisons during assembly. Further explanation of each parameter is found in the Phrap documentation [33].

Previously characterized repetitive sequences from soybean available at the time of assembly were included in prescreening during assembly (Marek et al. unpublished results) [39]. Quantification of assemblies was done using Vmatch for large-scale sequence matching (a large-scale global sequence alignment)[35]. This program returns the percent nucleotide identity as well as the start and stop position for each contig alignment to allow for the calculation of percent coverage. Only contigs that contained greater than 100 traces were included in the analysis.

Trace files from the soybean whole-genome shotgun sequencing effort were downloaded from the NCBI trace archive [72]. These files are reads all uploaded from August 9–10, 2006 (ti's range from 1397334945 – 1399236113) to for a total of 80,000 sequencing reads. To determine the sequence composition of the JGI-only assemblies, contigs contained greater than 15 traces were blasted against the nr database to assign a putative annotation. These contigs were assumed to represent what will be observed at a high frequency in the whole-genome assemblies.

## List of abbreviations

BAC – bacterial artificial chromosome; WGS – whole genome shotgun; SSR – simple sequence repeat; RFLP – restriction fragment length polymorphism; Ks – synonymous substitution; Ka – nonsynonymous substitution; Mya – million years ago; bp – base pair

## Competing interests

The author(s) declares that there are no competing interests.

## Authors' contributions

JAS designed this study, sequenced BACs, annotated BACs, designed primers for mapping of BACs, performed sequence alignments and divergence estimates, carried out all of the batch sequence assemblies and quantification of those assemblies and drafted the manuscript. JYL identified and sequenced BACs anchored by the RFLP clones A711 and participated in drafting the manuscript. SDS developed and set up the GmaxGDB database that was utilized for annotation of BACs and aided in the quantification of assemblies. IFVS, SD, JY and MO participated in sequencing of BACs. BAR coordinated sequencing of BACs and helped to draft the manuscript. RTN participated in annotating BACs. BES carried out sequencing of BACs. SAJ and RCS helped to design this study as well as draft the manuscript. All authors read and approved the final manuscript.

## Supplementary Material

Additional file 1 **Supplemental Figure 1**. VISTA identity plot between BACs GM_UMb001_24d13 and GM_UMb001_5f5. Each colored block represents a predicted gene structure from start to stop including introns with gray boxes between genes showing homoelogous relationships. The identity plots above and below each BAC structure show the nucleotide identity between each BAC based upon an annotation anchored global-pairwise alignment. The light purple boxes above each VISTA correspond to annotated exon positions. The GM_UMb001-24d13 selenium-binding gene appears shorter due to the coding region being in only exon 1; whereas the coding region of GM_UMb001-5f5 selenium-binding gene includes intronic sequence. Click here for file

Additional file 2 **Supplemental Figure 2**. VISTA identity plot between BACs gmw2-133d1 and gmw1-93l19. Each colored block represents a predicted gene structure from start to stop including introns with gray boxes between genes showing homoelogous relationships. The identity plots above and below each BAC structure show the nucleotide identity between each BAC based upon an annotation anchored global-pairwise alignment. The light purple boxes above each VISTA correspond to annotated exon positions. Click here for file

Additional file 3 **Supplemental Figure 3**. VISTA identity plot between BACs gmw1-103e11 and gmw1-5g16. Each colored block represents a predicted gene structure from start to stop including introns with gray boxes between genes showing homoelogous relationships. The identity plots above and below each BAC structure show the nucleotide identity between each BAC based upon an annotation anchored global-pairwise alignment. The light purple boxes above each VISTA correspond to annotated exon positions. The gmw1-5g16 RAD1-like gene is truncated relative to the gmw1-103e11 copy by a stop codon in the third exon. Both RAD1-like genes have complete EST support for gene structures. Similarly, the gmw1-5g16 galactinol synthase gene is truncated due to an EST supported alternative splicing event relative to the gmw1-103e11 copy. The gmw1-103e11 *A. thaliana*-like NAP gene covers only 5 of the 7 predicted exons with almost full EST support whereas the gmw1-5g16 copy covers all 7 exons with 100% EST support. Click here for file

Additional file 4 **Supplemental Figure 4**. VISTA identity plot between BACs gmw1-8g7 and gmw1-13o17. Each colored block represents a predicted gene structure from start to stop including introns with gray boxes between genes showing homoelogous relationships. The identity plots above and below each BAC structure show the nucleotide identity between each BAC based upon an annotation anchored global-pairwise alignment. The light purple boxes above each VISTA correspond to annotated exon positions. Click here for file

Additional file 5 **Supplemental Figure 5**. VISTA identity plot between BACs gmw1-57d24 and gmw1-58k3. Each colored block represents a predicted gene structure from start to stop including introns with gray boxes between genes showing homoelogous relationships. The identity plots above and below each BAC structure show the nucleotide identity between each BAC based upon an annotation anchored global-pairwise alignment. The light purple boxes above each VISTA correspond to annotated exon positions. A third BAC gmw1-27d20 is shown with homeologs to gmw1-57d24 and gmw1-58k3 but because this BAC is phase I (unordered contigs) no identity plots are show because the order of the contigs is unknown. Click here for file

Additional file 6 **Supplemental Table 1**. Contains homeolog-specific primer sequences used to identify BACs for sequencing. Both forward and reverse primers as well as their size and the BAC they identified are shown. Primers for BACs gmw1-52d3 and gmw1-74i13 are found in [8] and primer for gmw1-105h23, gmw1-15k6 and gmw1-11j16 are found in [19]. Click here for file

Additional file 7 **Supplemental Table 2**. Contains primers that amplify simple sequence repeats for mapping designed from homeologous BACs. Primers for BACs gmw1-52d3 and gmw1-74i13 are found in [8] and primer for gmw1-105h23, gmw1-15k6 and gmw1-11j16 are found in [19]. Click here for file
