# Supplementary figures and images for: Gene duplication and paleopolyploidy in soybean and the implications for whole genome sequencing
Source: BMC Genomics. 2007 Sep 19;8:330. doi: 10.1186/1471-2164-8-330 (PMC2077340; doi:10.1186/1471-2164-8-330)

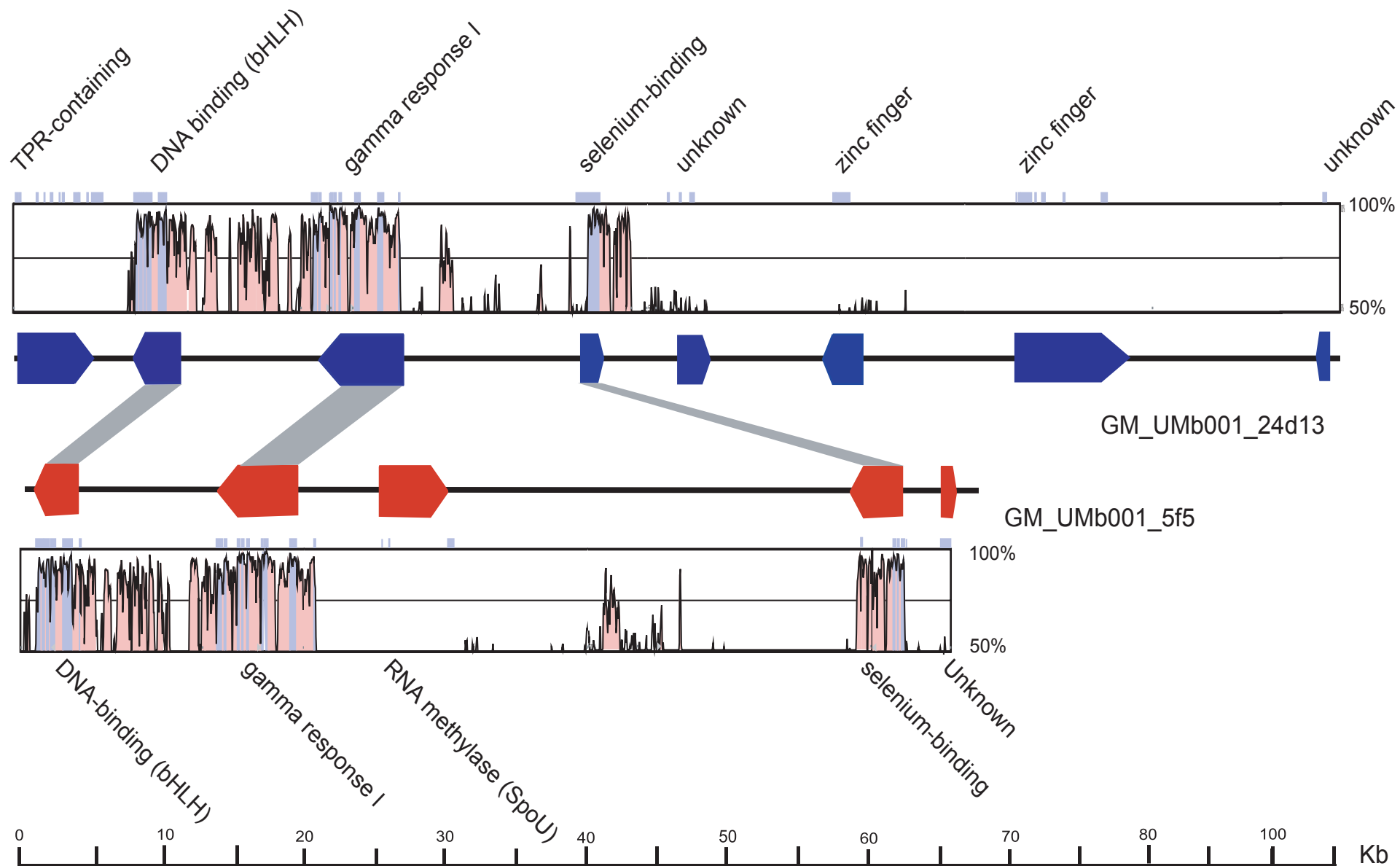

Supplement: Additional file 1 — Supplemental Figure 1. VISTA identity plot between BACs GM_UMb001_24d13 and GM_UMb001_5f5. Each colored block represents a predicted gene structure from start to stop including introns with gray boxes between genes showing homoelogous relationships. The identity plots above and below each BAC structure show the nucleotide identity between each BAC based upon an annotation anchored global-pairwise alignment. The light purple boxes above each VISTA correspond to annotated exon positions. The GM_UMb001-24d13 selenium-binding gene appears shorter due to the coding region being in only exon 1; whereas the coding region of GM_UMb001-5f5 selenium-binding gene includes intronic sequence. [file 1471-2164-8-330-S1.pdf]

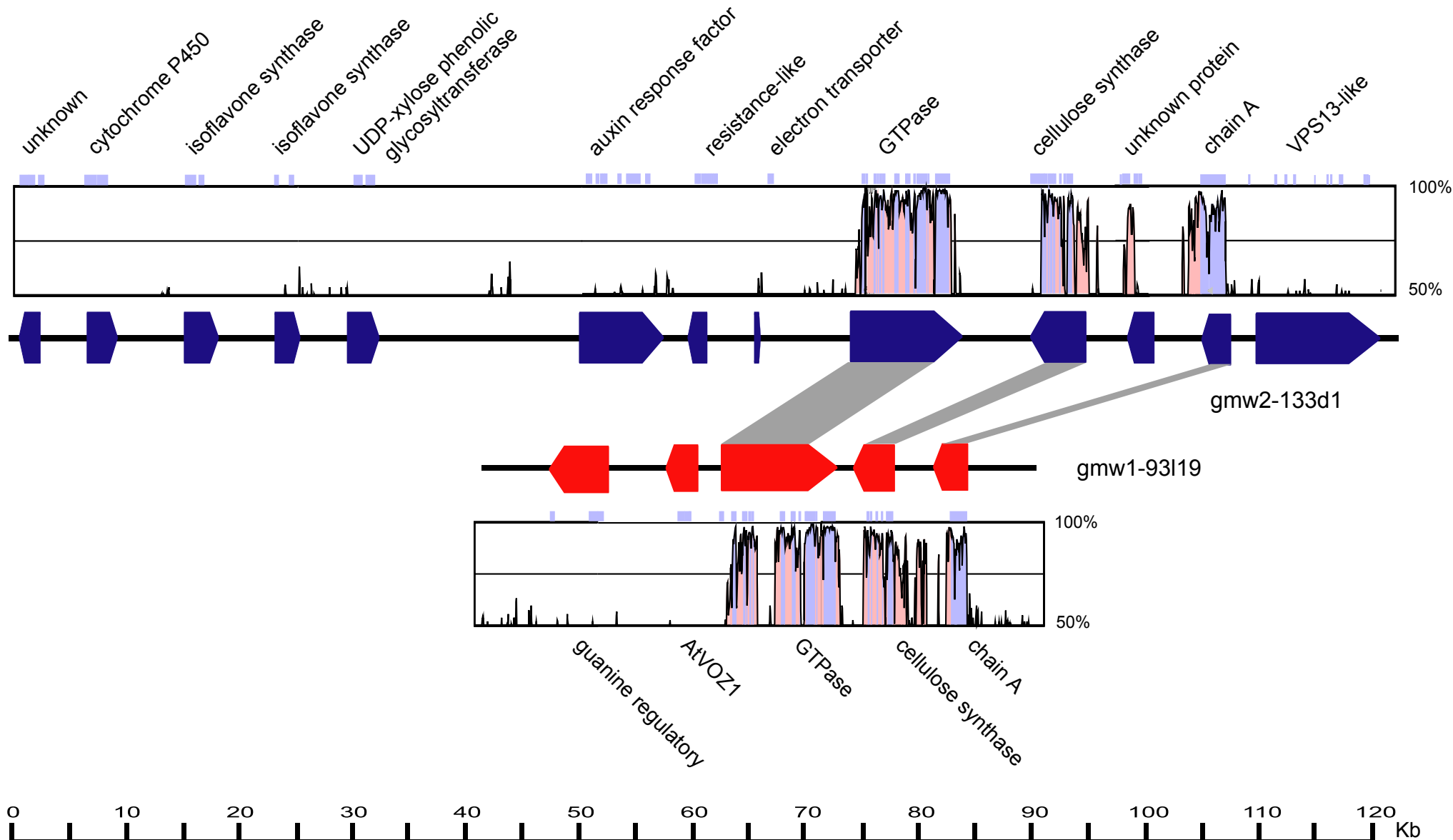

Supplement: Additional file 2 — Supplemental Figure 2. VISTA identity plot between BACs gmw2-133d1 and gmw1-93l19. Each colored block represents a predicted gene structure from start to stop including introns with gray boxes between genes showing homoelogous relationships. The identity plots above and below each BAC structure show the nucleotide identity between each BAC based upon an annotation anchored global-pairwise alignment. The light purple boxes above each VISTA correspond to annotated exon positions. [file 1471-2164-8-330-S2.pdf]

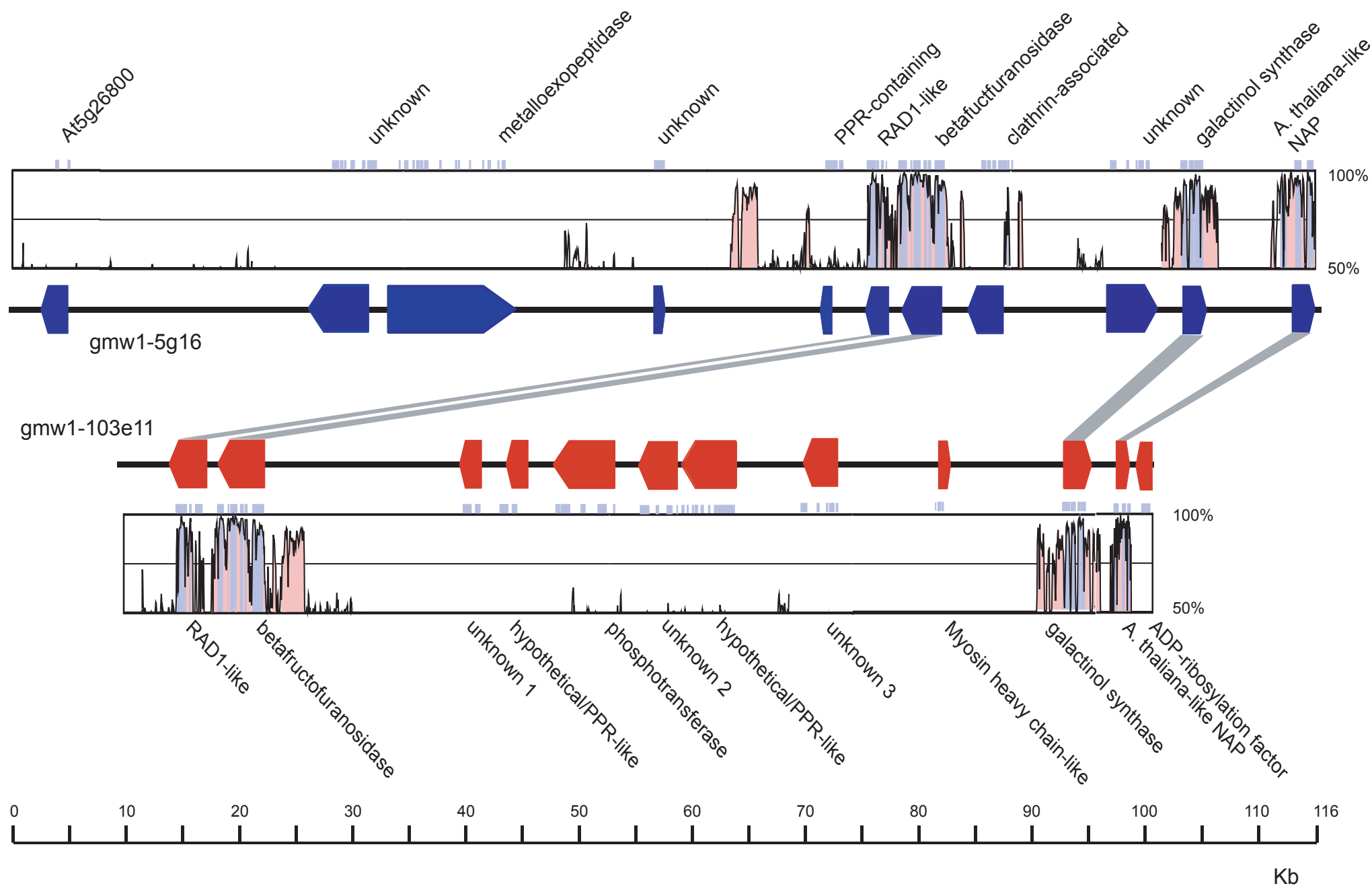

Supplement: Additional file 3 — Supplemental Figure 3. VISTA identity plot between BACs gmw1-103e11 and gmw1-5g16. Each colored block represents a predicted gene structure from start to stop including introns with gray boxes between genes showing homoelogous relationships. The identity plots above and below each BAC structure show the nucleotide identity between each BAC based upon an annotation anchored global-pairwise alignment. The light purple boxes above each VISTA correspond to annotated exon positions. The gmw1-5g16 RAD1-like gene is truncated relative to the gmw1-103e11 copy by a stop codon in the third exon. Both RAD1-like genes have complete EST support for gene structures. Similarly, the gmw1-5g16 galactinol synthase gene is truncated due to an EST supported alternative splicing event relative to the gmw1-103e11 copy. The gmw1-103e11 A. thaliana-like NAP gene covers only 5 of the 7 predicted exons with almost full EST support whereas the gmw1-5g16 copy covers all 7 exons with 100% EST support. [file 1471-2164-8-330-S3.pdf]

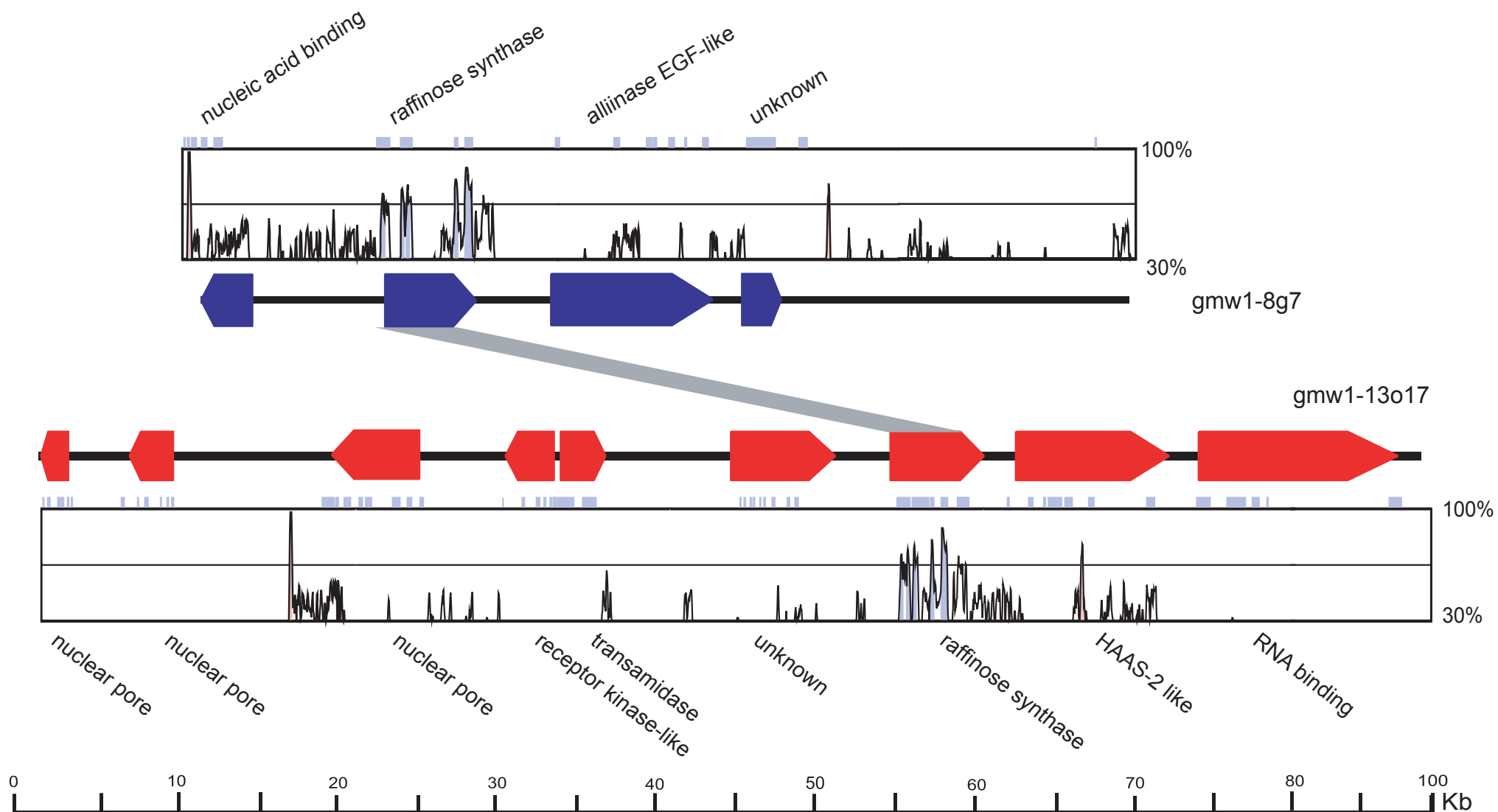

Supplement: Additional file 4 — Supplemental Figure 4. VISTA identity plot between BACs gmw1-8g7 and gmw1-13o17. Each colored block represents a predicted gene structure from start to stop including introns with gray boxes between genes showing homoelogous relationships. The identity plots above and below each BAC structure show the nucleotide identity between each BAC based upon an annotation anchored global-pairwise alignment. The light purple boxes above each VISTA correspond to annotated exon positions. [file 1471-2164-8-330-S4.pdf]

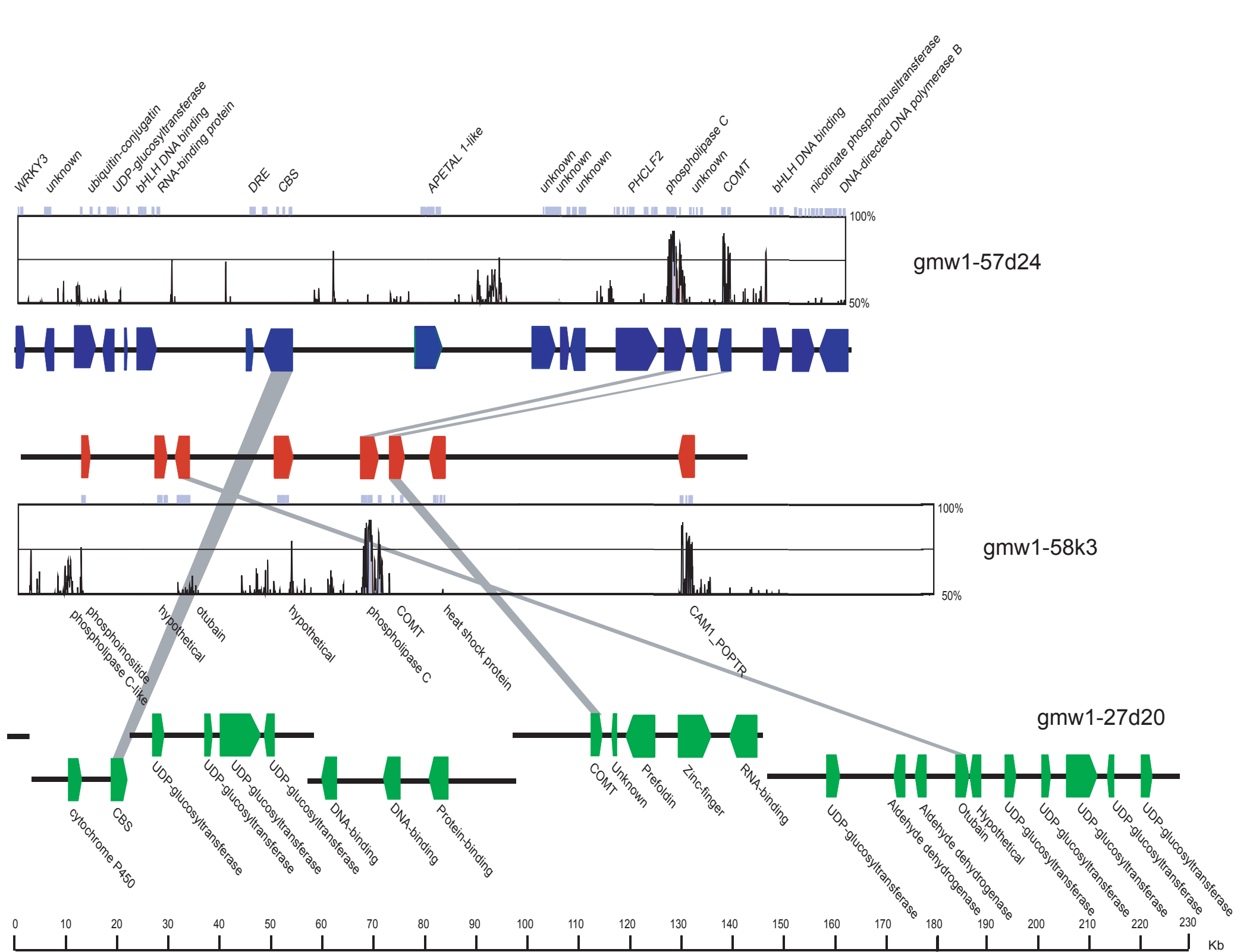

Supplement: Additional file 5 — Supplemental Figure 5. VISTA identity plot between BACs gmw1-57d24 and gmw1-58k3. Each colored block represents a predicted gene structure from start to stop including introns with gray boxes between genes showing homoelogous relationships. The identity plots above and below each BAC structure show the nucleotide identity between each BAC based upon an annotation anchored global-pairwise alignment. The light purple boxes above each VISTA correspond to annotated exon positions. A third BAC gmw1-27d20 is shown with homeologs to gmw1-57d24 and gmw1-58k3 but because this BAC is phase I (unordered contigs) no identity plots are show because the order of the contigs is unknown. [file 1471-2164-8-330-S5.pdf]
