# Supplementary material for: Gene duplication and paleopolyploidy in soybean and the implications for whole genome sequencing
Source: BMC Genomics. 2007 Sep 19;8:330. doi: 10.1186/1471-2164-8-330 (PMC2077340; doi:10.1186/1471-2164-8-330)
Supplement: Additional file 6 — Supplemental Table 1. Contains homeolog-specific primer sequences used to identify BACs for sequencing. Both forward and reverse primers as well as their size and the BAC they identified are shown. Primers for BACs gmw1-52d3 and gmw1-74i13 are found in [8] and primer for gmw1-105h23, gmw1-15k6 and gmw1-11j16 are found in [19]. [file 1471-2164-8-330-S6.pdf]

Supplemental Table 1. Homeolog specific primers to identify BACs

Primer name[a]	Primer sequence (5'-3')	Primer Length (bp)	Identified BAC[b]
Cellulose synthase 1U	CAT AAG CTC CTC GGT CAA G	19	gmw2-133d1
Cellulose synthase 1L	CCT TAT TCT CTC TCT ACT TTG	21	
Cellulose synthase 2U	ACA CTA CTA TTG TAT ATG ATG ATT	24	gmw1-93l19
Cellulose synthase 2L	TGG GCA AAG ATA GAC TCG TT	20	
Galactinol synthase 1U	CTC TGG TGG TTG CAG TGC TA	20	gmw1-5g16
Galactinol synthase 1L	GAA GGA GGT CAC GGT AAG TAG C	22	
Galactinol synthase 2U	TCT GAG GAA GGT GAA AAG CCT G	22	gmw1-103e11
Galactinol synthase 2L	GAG GTC ACG GTA GGT ATC CAG A	22	
COMT 1U	TGT CTA TCC AAG AGA GCA CG	20	gmw1-58k3
COMT 1L	GGC TTA ATG TAA TCC ATC AAT G	22	
COMT 2U	GGA GCT AAG AGA GTT GAC GGA	21	gmw1-57d24
COMT 2L	TCT CGA CCC TTG AAT CAA GTG	21	
COMT 3U	TAC CCA AGA GAA CCT GAA TCC A	22	gmw1-27d20
COMT 3L	CAG CAC GAA GTC CCT GTA GTA	21	
Raffinose synthase 1U	CAG CTT GTG GAT GGG CAA TTT C	22	gmw1-13o17
Raffinose synthase 1L	AAA CAC CCC AAG AAC TCC AGT G	22	
Raffinose synthase 2U	GAT GGG GAA TTT TAT TCA GCC G	22	gmw1-8g7
Raffinose synthase 2L	CAC CTC CTT GGC AAT TAA ATA G	22	

[a] Copy number was arbitrarily assigned for tracking primers, does not reflect what may be in the literature

[b] Corresponding BAC that the primer pair identified and was sequenced

[c] Size of PCR amplicon
