# Supplementary material for: Gene duplication and paleopolyploidy in soybean and the implications for whole genome sequencing
Source: BMC Genomics. 2007 Sep 19;8:330. doi: 10.1186/1471-2164-8-330 (PMC2077340; doi:10.1186/1471-2164-8-330)
Supplement: Additional file 7 — Supplemental Table 2. Contains primers that amplify simple sequence repeats for mapping designed from homeologous BACs. Primers for BACs gmw1-52d3 and gmw1-74i13 are found in [8] and primer for gmw1-105h23, gmw1-15k6 and gmw1-11j16 are found in [19]. [file 1471-2164-8-330-S7.pdf]

Supplemental Table 2. SSR primer sequences

Primer name[a]	Primer sequence (5'-3')	Primer Length (bp)
gmw2-133d1 U	AAG TAT GAT AAT TCT ACT AT	20
gmw2-133d1 L	GTT CAG ATA TGG AGA AGA GA	20
gmw1-93l19 U	CAT CCC TCC TAC CCA AAA CGC AA	23
gmw1-93l19 L	CAC AAC GCA CAA AAA CAA ACA CCC	24
gmw1-103e11 U	TGG CGC AAT ACA GGG GTT AGG A	22
gmw1-103e11 L	CCA AAC AAC CCC TTC AAT GTT AGA CG	26
gmw1-5g16 U	AGT CCA AAG TCT CAT CCT C	19
gmw1-5g16 L	CTG TCA CAC CAA TTC AAC CA	20
gmw1-58k3 U	ATA AAT ATA TCT TAT TTA ATG T	22
gmw1-58k3 L	GTT AGA CTA ATT AAC GAA GAG T	22
gmw1-57d24 U	GAC CTT CTC TTC ATA CAT CA	20
gmw1-57d24 L	GTG ACT ACA GGT TCG CCT TGA	21
gmw1-27d20 U	TAG GAT AAC ACA CTG TAG GAG	21
gmw1-27d20 L	CTA TAG CTG CTA TAG CTT TA	20
gmw1-13o17 U	CGC CCA ATT GTA CGC TTA GGG AT	23
gmw1-13o17 L	TGC ACC TCC ACT GTG ATT CCC	21

[a] BAC that the SSR was identified from and primer pairs designed from.
